# Determinants of thermal homeostasis in the preimplantation embryo: a role for the embryo’s central heating system?

**DOI:** 10.1007/s10815-024-03130-9

**Published:** 2024-05-08

**Authors:** Henry J. Leese, Roger G. Sturmey

**Affiliations:** 1 Centre for Biomedicine, Hull York Medical School, University of Hull, Hull, HU6 7RX UK; 2 Biomedical Institute for Multimorbidity, Hull York Medical School, University of Hull, Hull, HU6 7RX UK

**Keywords:** Thermal regulation, Embryo development, Mitochondrial function

## Abstract

A number of factors may impinge on thermal homeostasis in the early embryo. The most obvious is the ambient temperature in which development occurs. Physiologically, the temperature in the lumen of the female tract is typically lower than the core body temperature, yet rises at ovulation in the human, while in an IVF setting, embryos are usually maintained at core body temperature. However, internal cellular developmental processes may modulate thermal control within the embryo itself, especially those occurring in the mitochondria which generate intracellular heat through proton leak and provide the embryo with its own ‘central heating system’. Moreover, mitochondrial movements may serve to buffer high local intracellular temperatures. It is also notable that the preimplantation stages of development would generate proportionally little heat within their mitochondria until the blastocyst stage as mitochondrial metabolism is comparatively low during the cleavage stages. Despite these data, the specific notion of thermal control of preimplantation development has received remarkably scant consideration. This opinion paper illustrates the lack of reliable quantitative data on these markers and identifies a major research agenda which needs to be addressed with urgency in view of laboratory conditions in which embryos are maintained as well as climate change–derived heat stress which has a negative effect on numerous clinical markers of early human embryo development.

In this opinion paper, factors determining the maintenance of temperature in preimplantation embryos are examined, a topic which has assumed special significance due to the impact of heat stress on reproductive medicine associated with climate change and global warming [1]. Effects of excess heat on the early events of mammalian reproduction include diminished gamete and embryo development and viability, increased mitochondrial activity and associated production of reactive oxygen species and decreased offspring weight. These effects have been well-summarised for the human by Boni et al. [2] and in farm animals by Hansen [3].

The focus here is on physiological factors that may impinge on thermal homeostasis during the preimplantation stages of development, especially on the heat-generating capacity of mitochondria within the embryo, a topic which has attracted much less attention than the well-known involvement of mitochondria in other early cellular functions including apoptosis, [Ca^++^]i regulation, reactive oxygen species formation, redox status, metabolic regulation, maternal inheritance [4] and the provision of a *central signalling hub* [5].

Factors involved in thermal homeostasis in early embryos have been identified from a narrative review of the literature generated through a search of online databases to identify existing peer-reviewed literature on preimplantation embryos and somatic cells where appropriate. Pre-prints and the grey literature have been excluded.

## Ambient temperature in situ and impact on the development of the gametes and early embryo

Somewhat counterintuitively, the temperatures to which mammalian gametes and preimplantation embryos are exposed in situ (the ovarian follicle, oviduct and uterus) are *lower* than that of core body temperature, by ~ 1–2 °C. Values and temperature gradients for the different regions are summarised in the excellent account by Hunter [6] which includes a discussion of the anatomical origin of these temperature differences and their implications for gamete physiology. A fine, more recent account is given by Ng et al. [7].

Deriving from classical works from Van’t Hoff [8] and Arrhenius [9] at the turn of the last century, it has long been known that the rate of a chemical reaction is intrinsically dependent on temperature. For example, the major impact of a reduction in ambient temperature will be a diminished rate of metabolic reactions [10]. Various mathematical models have been proposed to describe this relationship, with ‘Q_10_’ perhaps being the most established. This describes the extent to which the rate of reaction depends on temperature across a fixed range, usually 10 °C [11]. Typically, the Q10 for metabolic rate is around 2. This means that for a temperature shift of 10 °C, the metabolic rate will double or halve, and for a change of ~ 1 °C, i.e. the minimum order of magnitude being considered here, 10%. In other words, the metabolism of the early embryo in the reproductive tract could be around at least 10% lower than it would be where it maintained at core body temperature (37–39 °C depending on the species). This may contribute to differences seen in metabolic activity between in vivo–derived and in vitro culture embryos (for example see [12]). Such a reduction, by analogy with numerous studies in cultured somatic cells, will reduce oxidative stress, especially reactive oxygen species (ROS) formation [13, 14], more than 95% of which are generated by mitochondrial oxidative metabolism [15]. It should be noted that while excess ROS are potentially harmful to many cell functions inducing DNA damage and compromising gene expression, they also perform essential signalling roles required in the embryo for components of pronuclear formation, first cleavage, cell proliferation and apoptosis [15].

## Rise in core body temperature that accompanies ovulation

It is widely accepted that body temperature in women changes throughout the menstrual cycle [16]. For a review of regulatory factors, see Baker et al. [17]. Prior to ovulation, the core body temperature lies typically between 35.5 and 36.6 °C, whereas 24 h after ovulation, it rises to between 36.1 and 37.2 °C, an average increase of 0.5–0.6 °C, though with considerable variation between individuals. It is of particular interest that this elevated body temperature persists for about 7 days [16], i.e. spanning the process of fertilisation and the formation of the mature blastocyst and may, in the absence of other factors, lead to an increase of 5–6% in metabolic rate throughout this period of development, according to the Q_*10*_ concept, and presuming that the change in basal body temperature is reflected in the female reproductive tract.

It is important to discover whether a modest increase or decrease in temperature impacts on the reproductive tract; for example, Matsuzuka et al. [18] reported that heat stress in mice (an ambient temperature of 35 °C for 12, 24 or 36 h) was associated with a linear increase in ROS formation in the oviduct. Whether this leads to an increase in ROS content in the embryo and the extent to which this has a positive or negative effect on embryo physiology is difficult to ascertain in view of the confounding factors being considered in this review.

Observations such as these are particularly timely when considering energy metabolism in the early embryo. We have a mature understanding of the importance of energy metabolism during the preimplantation phase, not least in the generation of metabolic energy [19]. However, it is becoming increasingly apparent that metabolic processes play intrinsic roles in regulating wider aspects of development, including gene expression, epigenetic regulation and genome activation [20]. Importantly, our knowledge of such factors is based almost entirely on studies in vitro—even when considering in vivo embryos, metabolic measurements have been made after they have been retrieved from the physiological compartment of growth and placed in in vitro conditions. Almost universally, embryos are maintained in vitro under temperatures based on that of the core body temperature of the host species: 37 °C in the mouse, 37 °C in the human, 39 °C in the bovine. This means that the current understanding of embryo metabolism may not reflect fully the situation in situ; indeed, reports are emerging that highlight significant differences in the metabolic profile of embryos produced in vitro compared to naturally conceived counterparts [12]. Moreover, it prompts further consideration of the role of subtle temperature fluctuations during preimplantation development, especially within the context of altered environmental temperatures and increased thermal stress placed on humans and animals.

## Developmental and functional aspects of preimplantation development which may mitigate effects of heat stress

The energy requirements of developmental processes occurring in the embryo during the preimplantation stage are relatively low [19] and are largely satisfied through mitochondrial oxidative metabolism, with a minor, but important contribution from aerobic glycolytic production of lactate [20]. Thus, while the fully grown mouse oocyte contains 100,000–200,000 individual mitochondria, they appear ‘immature’ with poorly developed cristae [21]. Overall, oxygen consumption by oocytes and all stages of development until the blastocyst are low and relatively constant [22], a pattern that appears broadly conserved across a variety of mammals [23].

Uniquely, in the preimplantation embryo, while the nuclear material is duplicated during the cleavages of the zygote, the cellular contents (including the mitochondria) are not. A net result of this lack of increase in cell mass is that the two most energy-demanding, heat-producing, metabolic processes protein synthesis and plasma membrane sodium pump (Na + , K + , ATPase) remain low. Together, these two processes comprise approximately two-thirds of the energy budget of somatic cells. As the number of cells increases markedly with blastocyst formation, so does the amount of plasma membrane per embryo. This is accompanied by elevated activity of the overall Na + , K + , ATPase activity which coincides with an increase in protein content in the blastocyst as the embryo initiates net growth for the first time. These processes are driven largely by a sharp rise in mitochondrial oxidative phosphorylation [22]. However, in terms of heat generation, the survival of the stages of preimplantation development is likely to benefit from their being relatively quiescent metabolically.

## Further factors which impact thermal homeostasis in the oocyte and preimplantation embryo

### Mitochondrial heat production

One factor, which, to the best of our knowledge, has not been considered in the present context, is the generation of heat by early embryos themselves. Such heat, as in all homeotherms, largely originates in the mitochondria and is a consequence of oxidative phosphorylation as already indicated. There is also strong evidence for the utilisation of endogenous lipid as an energy substrate by early embryos particularly in the human and domestic animals [24] which will yield over twice the amount of heat than from carbohydrate or protein oxidation. The efficiency of oxidative phosphorylation in terms of ATP formation in mature mitochondria is ~ 40% with the remaining ~ 60% being released as heat by basal and inducible proton leak; for excellent reviews, see Beignon et al. [25] and Bertholet and Kirichok [26], values which, however, can differ in different cells and metabolic states. For example, Muller et al. [27] reported a figure of 20% for the proton leak of in vitro–produced bovine preimplantation embryos measured as oxygen consumption insensitive to oligomycin. Significantly, as well as generating heat, the proton leak reduces the production of ROS and therefore offers a protective mechanism against oxidative stress.

About 10 years ago, it was suggested that mitochondria might themselves have a higher temperature than their intracellular surroundings. To test this idea, Okabe et al. [28] used heat-sensitive fluorescence probes in a fibroblast-derived cell line and reported that localised heat production did occur around mitochondria and that the temperature in the nucleus of fibroblast-derived cells was also higher than in the cytoplasm. Precise values for such temperature differences in this and similar studies were tentative but thought to be ~ 0.5–1 °C.

A startling observation was reported in 2018 in *PLoS Biol* by Chrétien et al. [29] with the title ‘Mitochondria are physiologically maintained at close to 50 °C’, a temperature at which proteins would denature! This report was the subject of a critical, though constructive, commentary in the same issue of the journal by Lane [30] entitled ‘Hot mitochondria?’ which discussed the validity of the method used (the thermosensitive dye Mito Thermo Yellow), the structure of mitochondria, not as simple membrane-bound spheres but comprising a parallel arrangement of the cristae on the inner mitochondrial membrane, as a series of membranes, termed ‘radiators’ by Lane. This is a most appropriate analogy, which in lay terms may be envisioned as early embryos having their own central heating system (mitochondria) and radiators within them (mitochondrial cristae).

Macherel et al. [31] in a comprehensive review, entitled ‘The conundrum of hot mitochondria’ emphasised the numerous uncertainties surrounding this question. Three such problems stand out: first is the technological difficulty in making such measurements; second, the nature of heat conductivity in individual cells; third is the question of ‘proton leak’, when ATP generation is uncoupled from substrate oxidation such that all the energy from electron transfer is released as heat as considered below.

Methodological problems and heat conductivity have also been explored in a fascinating study by Song et al. [32] who reported that thermal conductivity differed within single human cells (HeLa, MCF-7 and MCF-10A) with heat transfer greater at the periphery than the centre. This may be pertinent especially in the context of the mammalian oocyte and zygote, which have a large diameter, compared to somatic cells. Moreover, responses were sensitive to the ambient temperature which led Song et al. to put forward the radical hypothesis that warm-blood animal cells could adjust their thermal conductivity according to the ambient temperature to promote their own cellular heat balance, akin to the whole body. Such a phenomenon would obviously be of great interest in the case of the semi-autonomous preimplantation embryo, but Song et al. cautioned that the *relationship between the changes of cellular thermal conductivity and the cellular response to changes in ambient temperature undoubtedly demands in-depth and comprehensive studies in both biophysics and cell biology*. As concluded by Kruglov et al. [33] in a recent review entitled ‘Warm Cells, Hot Mitochondria: Achievements and Problems of Ultralocal Thermometry’, ‘currently, it is difficult to name a good sensor for measuring temperature in mitochondria’. El-Gammal et al. [34] have provided an excellent overview of this topic which emphasises the importance of reporting the environmental conditions under which measurements are made and the potential ranges of mitochondrial temperature, and a recent article by Moreno-Loshuertos et al. [35] claims that an incubation temperature above 43 °C leads to the degradation of respiratory complexes in human cultured cells, adding to the complexity of intracellular thermoregulation and homeostasis.

### The intracellular movement of mitochondria

Mitochondrial motility has been well-summarised recently by Harrington et al. [36]. There is considerable intracellular movement related to mitochondrial fission and fusion mediated by cytoplasmic motor proteins which transport mitochondria along cytoplasmic filaments. There may be distribution to sites where increased energy is required, and some intercellular transport of mitochondria. With regard to thermal homeostasis, such mechanisms could serve to buffer the high local temperatures generated in the immediate vicinity of mitochondria, a notion proposed for the local accumulation of ATP, by Van Blerkom [37], but more research is required to equate these ideas to heat distribution.

## Conclusion

### Thermal homeostasis in the mammalian preimplantation embryo

This brief survey has documented potential determinants of the thermal status of the preimplantation embryo. The information has not been given in precise quantitative terms because such data do not exist; rather, this review of the literature has revealed an extensive research agenda which needs to be addressed with urgency, especially as the global ambient temperature will continue to increase for the foreseeable future. Research is needed to

- *Quantify the contribution of the factors which determine thermal homeostasis during the various stages of preimplantation development.*
- *Draw up a model which combines the various factors under different physiological conditions and incorporates intrinsic cellular defence mechanisms to counteract heat (not considered in this Opinion piece).*
- *Chart the response of the model to environmental, notably, heat stress.*
- *Integrate the conclusions to provide sound advice to mitigate heat stress during conception *in vivo* and *in vitro*.*
- *Repeat this analysis for the gametes in their environments in the male and female reproductive tracts.*
- *Explore the extent to which, if at all, eggs and early embryos, and by implication their mitochondria, have the capacity to self-regulate their heat production, i.e. act like thermostats.*

In proposing these research aims, we do not underestimate the technical difficulties involved, notably in the measurement of intracellular and subcellular temperatures, as we have referenced [31–33]. However, it has not been our intention to provide detailed experimental protocols; rather, to hope that if our paper stimulates interest in thermal homeostasis in the gametes and early embryo, it will prompt others to refine existing techniques and develop new ones. It may be of advantage that the egg is the largest cell in the female mammal and provides a much larger experimental target than the average somatic cell.

There can be some optimism that the findings of such research could ultimately be applied to address problems of elevated ambient temperature during the early events of human conception and throughout pregnancy. Such optimism comes from the substantial literature on the reproductive physiology of heat-stressed farm animals, especially the dairy cow where the annual economic loss due to heat stress for such animals in the USA is ~ $1.5B. Hansen [3], Mietkiewska et al. [38] and Khan et al. [39] have provided excellent accounts of such research and the prospects of it leading to new strategies to mitigate the effects of overheating. With regard to the human, it is well-recognised that environmental conditions during the periconception period can influence the health of the conceptus and newborn, and potentially, the offspring in later life. Preimplantation development is particularly susceptible to environmentally induced perturbations leading to impaired future health [40], and it is critical to ensure that thermal aspects of gamete and early embryo physiology are well-defined and reflected in culture conditions. Clearly, this is a ‘hot topic’ for future research.

## Data Availability

No data were generated or reported in this manuscript.
